# EP300 promotes ferroptosis via HSPA5 acetylation in pancreatic cancer

**DOI:** 10.1038/s41598-023-42136-8

**Published:** 2023-09-11

**Authors:** Yuan Wang, Yang Liu, Cong Wang, Rui Kang, Daolin Tang, Jiao Liu

**Affiliations:** 1 DAMP Laboratory, Third Affiliated Hospital of Guangzhou Medical University, Guangzhou, 510150 Guangdong China; 2 Guangzhou Municipal and Guangdong Provincial Key Laboratory of Protein Modification and Degradation, Guangzhou Medical University, Guangzhou, 510150 Guangdong China; 3 Department of Surgery, UT Southwestern Medical Center, Dallas, TX 75390 USA

**Keywords:** Cancer therapy, Cell death

## Abstract

Ferroptosis is a form of regulated cell death characterized by oxidative injury-induced lipid peroxidation. However, the detailed protein post-translational modification regulatory mechanism of ferroptosis remains largely unknown. Here, we report that E1A binding protein P300 (EP300) acetyltransferase promotes ferroptosis in human pancreatic ductal adenocarcinoma (PDAC) cells via the acetylation of heat shock protein family A (Hsp70) member 5 (HSPA5), also known as GRP78 or BIP) on the site of K353. Acetylated HSPA5 loses its ability to inhibit lipid peroxidation and subsequent ferroptotic cell death. Genetic or pharmacological inhibition of EP300-mediated HSPA5 acetylation on K353 increases PDAC cell resistance to ferroptosis. Moreover, histone deacetylase 6 (HDAC6) limits HSPA5 acetylation and subsequent ferroptosis. Collectively, these findings not only identify regulatory pathways for HSPA5 acetylation during ferroptosis, but also highlight promising strategies to increase ferroptosis sensitivity in PDAC cells.

## Introduction

Pancreatic ductal adenocarcinoma (PDAC) accounts for 85% of pancreatic tumors and has a very poor prognosis with a 5-year survival rate of only 10%^1^. According to the latest data from the American Cancer Society, there will be about 62,210 new cases of pancreatic cancer in 2022, including about 49,830 deaths, which is expected to become the second leading cause of death from digestive system cancers^2^. Genetically, the progression of PDAC is primarily driven by KRAS mutations^3^. Therapeutically, this tumor responds poorly, mainly due to drug resistance^4^. There is an urgent need to further elucidate the pathogenesis of PDAC and find new ways to kill tumor cells.

Ferroptosis is a form of regulated cell death discovered by screening small-molecule compounds to selectively deplete RAS-mutant cancer cells^5^. Iron accumulation and increased lipid peroxidation are central events that induce ferroptosis^6^. Different from apoptosis and necroptosis, activation of caspase and mixed lineage kinase domain like pseudokinase is not required for the induction of ferroptosis^7^. Under different conditions, glutathione peroxidase 4 (GPX4), ferroptosis suppressor protein 1 (FSP1), and dihydroorotate dehydrogenase (DHODH) protect cells against ferroptosis by scavenging lipid peroxidation^6^. Conversely, an increased lipid supply, especially polyunsaturated fatty acids, increases susceptibility to ferroptosis^8^.

Lysine acetylation (Kac) is an important reversible and dynamic protein post-translational modification (PTM). Non-histone acetylation may modulate protein function including altering its conformation, stability, hydrophobicity, localization, or blocking its capacity to accept other post-translational protein modifications^9–11^. E1A binding protein P300 (EP300) and its closely related analogue cAMP response element-binding protein (CREBP) are widely expressed transcriptional coactivators and acetyltransferases of major lysine residues. EP300/ CREBP has different pro-death or anti-death effects in different tumor types with different genetic backgrounds^12^. In contrast, the activation of histone deacetylase (HDAC) family can limit protein acetylation^13^. Despite these advances, few studies have reported the effect of protein acetylation modifications on cancer cell ferroptosis.

HSPA5 is a member of the molecular chaperones expressed primarily in the endoplasmic reticulum (ER). The elevation of HSPA5 expression under various ER stress conditions suggests the involvement of HSPA5 in enhanced cell survival. Some.

studies have also found that HSPA5 plays an important role in cancer development and resistance^14–16^. Specifically, during ER stress, HSPA5 is titrated away from the sensors due to its ability to bind misfolded proteins with higher affinity, leading to activation of the ER stress sensors^17^. Our recent studies have reported that HSPA5 mediates ferroptosis resistance in PDAC cells by maintaining the stability of the GPX4 protein^15^. Here, we demonstrate that EP300-mediated acetylation of HSPA5 increases its antiferroptotic function by blocking GPX4. We further identified K353 as the direct acetylation site of HSPA5 responsible for ferroptosis sensitivity.

## Materials and methods

### Reagents

RSL3 (S8155), erastin (S7242), C646 (S7152), MG-132 (S2619), Bafilomycin A1 (S1413) were purchased from Selleck Chemicals. Trichostatin A (HY-15144) was obtained from MedChemExpress. The antibody to EP300 (sc-48343) was obtained from Santa Cruz. The antibodies to HSPA5 (11,587–1-AP) and TUBB (10,094–1-AP) were obtained from Proteintech. HDAC6 was obtained from Affinity (A5021). Acetylated-Lysine was obtained from Cell Signaling Technology (9814). The antibody to GPX4 was obtained from Abcam (ab125066).

### Cell culture

Human PDAC cell lines (PANC1, SW1990, MIA PaCa-2, ASPC1 and CFPAC1) were purchased for the American Type Culture Collection. These cells were cultured in Dulbecco's Modified Eagle Medium (C11885500BT, Gibco). supplemented with 10% heat-inactivated fetal bovine serum (42Q1074K, Gibco) and 1% penicillin and streptomycin (15,070–063, Thermo Fisher Scientific) at 37 °C and 5% CO_2_. All the cell lines were verified by short tandem repeat profiling, and the conventional mycoplasma test was negative for contamination.

### qPCR analysis

Total RNA was extracted and purified from cultured cells using the Cell Total RNA Isolation Kit (RE-03112, ForeGene). First-strand cDNA was synthesized from 1 μg of RNA using the PrimeScript RT Master Mix (RR036A, Takara)^18^. Then cDNA from various cell samples was amplified by real-time quantitative polymerase chain reaction (qPCR) with pre-designed primers from OriGene using a CFX96 touch real-time PCR detection system (Bio-Rad). Optimal dilution and melting curves were utilized to ensure the specificity of amplified production for each primer set. All expressions were calculated using the 2^-ΔΔCt^ method.

The primers were used as below:

Human *EP300*: 5′-ACCAGGAATGACTTCTAGTTTGA- 3′ and 5′-GGGTTTGCCGGGGTACAATA- 3’; Human *18SrRNA*: 5′-GGAGTATGGTTGCAAAGCTGA- 3′ and 5′-ATCTGTCAATCCTGTCCGTGT- 3′.

### Cell viability assay

The level of cell viability was assayed using a CCK8 kit (B34302, Bimake) according to the manufacturers protocol. Briefly, cells were cultured in 96-well plates (8000–10,000 cells per well) and treated with RSL3 or erastin for 24 h. For each well of the plate, the medium was replaced with 100 µl of fresh medium containing 1 µl of CCK8 solutions. The culture was then returned to the cell incubator for 30–60 min, and the absorbance value was measured at 450 nm. The absorbance at 450 nm was proportional to the number of living cells in the culture.

### Cell death assay

The Hoechst 33,342/propidium iodide (PI) cell death assay kit (BB-4131-1, BestBio) was used to assay cell death according to the manufacturer’s protocol. In brief, cells (3 × 10^5^/well) were seeded in 6-well cell culture plates and incubated in a humidified atmosphere of 5% CO_2_ at 37 °C. After 24 h, the cells were then treated with RSL3 for 24 h and stained with Hoechst33342 and PI. Morphological changes were examined by a fluorescence microscope at × 20 magnification.

### MDA assay

The relative MDA concentration in cell lysates was assessed using a lipid peroxidation MDA assay kit (S0131S, Beyotime) according to the manufacturer’s instructions. Briefly, the MDA in the sample reacted with thiobarbituric acid (TBA) to generate an MDA-TBA adduct. The MDA-TBA adducts were quantified colorimetrically (OD = 532 nm) or fluorometrically (Ex/Em = 532/553 nm). MDA levels were expressed as µmol/mg of protein. A malondialdehyde (MDA) molecule is co-heated with two thiobarbituric acid (TBA) molecules under acidic conditions to form a pink complex. The material has a maximum absorption peak at wavelength 532 nm. It can be determined by spectrophotometry. At Ex/Em = 532/553, the microdetermination can be made by fluorescence method.

### Lipid ROS assay

The lipid ROS level was analyzed using a BODIPY 581/591 C11 probe (D3861, Thermo Fisher Scientific). Cells were seeded at a density of 3 × 10^5^ per well in 6-well plates and grown for 24 h. Cells were treated with RSL3 for 24 h. The culture medium was replaced with a 2 ml medium containing 2 μM BODIPY 581/591 C11 of and cells were returned to the cell culture incubator for 30 min. Cells were harvested in 1.5 ml tubes and washed twice with ice phosphate-buffered saline (PBS) to remove the excess probe. After resuspending the cells in 400 ml PBS, the cell suspension was analyzed by flow cytometry to check the level of lipid ROS in the cells. A minimum of 10,000 cells were analyzed per condition.

### Iron assay

The intracellular iron level was measured using the Phen Green SK probe (P14313, Thermo Fisher Scientific) according to the manufacturer’s instructions. Briefly, cells were seeded at a density of 3 × 10^5^ per well in 6-well plates and grown for 24 h. Cells were treated with RSL3 for 24 h. The culture medium was replaced with 2 ml ice-cold PBS containing a 5 μM Phen Green SK probe and cells were put into the 37℃ incubator for 10–30 min. The fluorescence intensity changes were examined by a fluorescence microscope at × 20 magnification.

### Western blot

Western blots were performed as previously described ^19^. In brief, proteins in the cell lysate or supernatants were resolved on 7.5% and 10% polyacrylamide gel electrophoresis gels and transferred to a polyvinylidene difluoride membrane. After blocking with 5% milk at room temperature for 2 h, the membrane was incubated overnight at 4 °C with various primary antibodies. After incubation with peroxidase-conjugated secondary antibodies for 1 h at room temperature, the signals were visualized using SuperSignal West Pico PLUS Chemiluminescent Substrate (34,095, Thermo Fisher Scientific) and by using the ChemiDoc Touch Imaging System. All raw data is displayed in the supplementary information (Supplementary Data 4).

### RNA interference and gene transfection

The transfection of shRNA, siRNA, or cDNA was performed with Lipofectamine 3000 according to the manufacturer’s protocol. For the transfection of shRNA, 293 FT cells were used to produce high-titer lentiviral particles, and the virus-containing medium was harvested 48 h after transfection. RNAi was performed using lentiviral transduction as previously described^20^. Puromycin (0.5–10 μg/ml, A1113802, Thermo Fisher Scientific) and G418 (0.1–1 mg/ml, SG8160, Solarbio) were used for the selection of transduced cells. The EP300 mutants with K353R and K353Q deletion were generated using the Mut Express II Fast Mutagenesis Kit V2 (C214-01, Vazyme). Other commercial shRNA, siRNA, and cDNA sources are listed in Table S1.

### Immunoprecipitation analysis

Cells were lysed at 4 °C in ice-cold RIPA buffer (P0013D, Beyotime), and cell lysates were cleared by brief centrifugation (15,000 rpm, 15 min). Concentrations of proteins in the supernatant were determined using the BCA assay. The samples containing equal amounts of proteins were precleared with protein A agarose beads (4 °C, 2 h) before immunoprecipitation. After incubation with a variety of irrelevant IgG or specific antibodies, the mixture was gently shaken with protein A agarose beads for 2 h or overnight at 4 °C. Following incubation, agarose beads were washed extensively with PBST (0.5%Triton X-100 + PBS), and proteins were eluted by boiling in a 1 × loading buffer.

### Colony formation assay

For the colony formation assay, cells were seeded in 6 cm dishes at a density of 0.5 × 10^3^ per well and were treated with RSL3 for 24 h. Colonies were formed by trypsinizing, counting, and re-plating cells in appropriate dilutions in 6-well plates. After 14 days of incubation, colonies were fixed and stained with a mixture of methanol and 0.5% crystal violet for 30 min.

### Statistical analysis

Data are presented as mean ± SD of three independent experiments except where otherwise indicated. Statistics were calculated with GraphPad Prism 8.4.3. A standard two-tailed unpaired Student’s *t* test or one-way ANOVA was used for statistical analysis. A *P* value of < 0.05 was considered statistically significant.

## Results

### EP300 is upregulated during ferroptosis

**Figure 1:**
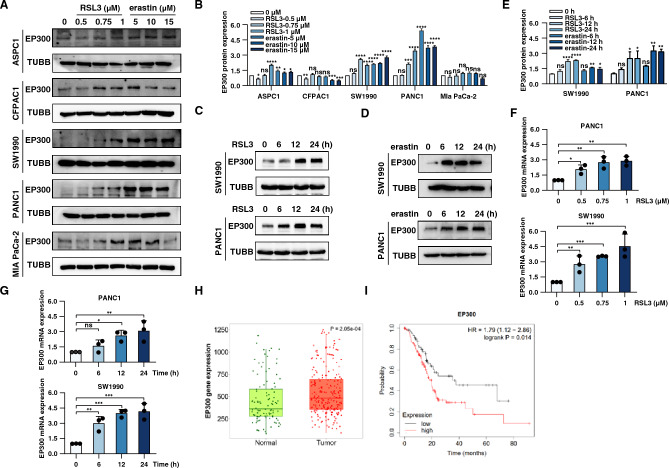
EP300 is upregulation during ferroptosis. (**A**–**B**) Western blot analysis of EP300 protein expression in ASPC1, CFPAC1, SW1990, PANC1 and MIA PaCa-2 cells treated with RSL3 (0.5, 0.75 and 1 μM) or erastin (5, 10 and 15 μM) for 24 h. (**C**-**E**) Western blot analysis of EP300 protein expression in PANC1 and SW1990 cells following treatment with RSL3 (1 μM) or erastin (10 μM) for 0–24 h. (**F**) qPCR analysis of EP300 gene expression in PANC1 and SW1990 cells following treatment with RSL3 (0.5, 0.75, and 1 μM) for 24 h. (**G**) qPCR analysis of EP300 gene expression in PANC1 and SW1990 cells following treatment with RSL3 (1 μM, 0–24 h). (**H**) EP300 gene expression in normal tissues and pancreatic cancer cells. Date from http://tnmplot.com/analysis/. (**I**) The clinical significance of EP300 expression in overall survivals in PDAC patients was evaluated in website http://kmplot.com/analysis/.

Our previous screening study found that the mRNA expression of *EP300* was increased after the treatment with RSL3 or erastin in PANC1 cell line^21^. Therefore, we continued to investigate whether EP300 protein expression was increased during ferroptosis. Western blot analysis revealed that RSL3 or erastin dose- and time-dependently increased EP300 expression in PANC1 and SW1990 cells after the treatment with RSL3 or erastin (Fig. 1A–E). qPCR analysis further confirmed that RSL3 dose and time-dependently increased EP300 mRNA expression (Fig. 1F–G). Notably, a recent genome-wide CRISPR screen in 786-O cells also identified EP300 as a positive and top regulator of ML210-induced ferroptosis^22^.

Next, we analyzed the relationship between EP300 expression and survival in PDAC patients using the public TCGA database. This bioinformatics analysis found that the expression of EP300 in pancreatic cancer tissues was higher than that in normal adjacent tissues (Fig. 1H). Kaplan–Meier tumor-free survival analysis further revealed that the overexpression of EP300 reduced PDAC the survival rate of patient (Fig. 1I). This suggests that the role of EP300 in promoting or suppressing tumors depends on tumor types.

### EP300 positively regulates ferroptosis

**Figure 2:**
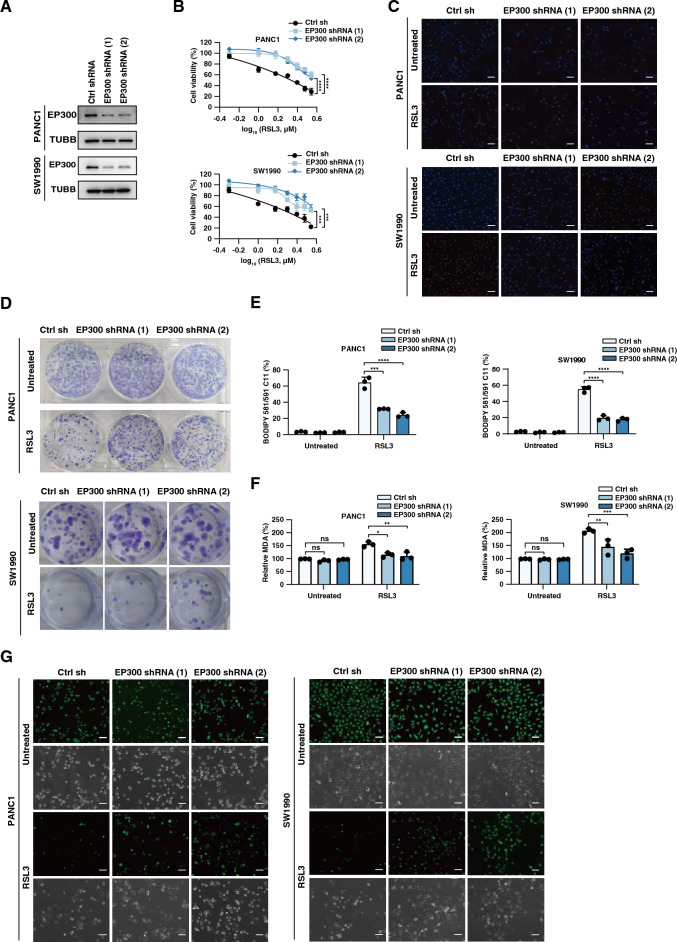
EP300 positively regulates ferroptosis. (**A**) Western blot analysis of EP300 protein expression in EP300-knockdown PANC1 and SW1990 cells. (**B**) Measuring cell survival by cell count kit 8 following treatment with RSL3 (1 μM) for 24 h. (**C**) Cell death by PI/hoechst33342 staining in the indicated cells following treatment with RSL3 (1 μM) for 24 h. Scale bar: 100 μm. (**D**) Colony formation assay in indicated PDAC cells following treatment with RSL3 (1 μM) for 24 h; then an equal amount of surviving cells was re-cultured in 6-well plates for 14 days. (**E**) The lipid ROS level was assessed by flow cytometry using a C11-BODIPY probe in indicated cells following treatment with RSL3 (1 μM) for 24 h. (**F**) Measuring oxidative stress using an MDA assay kit in indicated cells following treatment with RSL3 (1 μM) for 24 h. (**G**) Cellular iron accumulation level was assayed by PGSK (Phen Green SK) probe, Fluorescence intensity was observed under the fluorescence microscope. Scale bar: 100 μm.

To determine whether EP300 is a positive regulator of ferroptosis in PDAC cells, we used specific shRNAs to inhibit EP300 expression in PANC1 and SW1990 cells. Western blots confirmed that EP300 protein expression was significantly down-regulated after the transfection of EP300 shRNA (Fig. 2A). Cell viability assay showed that the knockdown of EP300 decreased the sensitivity of PANC1 and SW1990 cells to RSL3-induced ferroptosis (Fig. 2B). Consistently, EP300 knockdown reduced RSL3-induced cell death in PANC1 and SW1990 cells as detected by PI staining (Figs. 2C, S1A). Colony formation assays further demonstrated that the knockdown of EP300 decreased RSL3-induced tumor growth suppression (Figures D, S1B). Given that lipid peroxidation is an important signaling event for ferroptosis ^23^, we assayed the levels of lipid ROS, MDA, and iron. The knockdown of EP300 decreased RSL3-induced the production lipid ROS (Fig. 2E), MDA (Fig. 2F), and iron (Figs. 2G, S1C) in PANC1 and SW1990 cells.

To further confirm the role of EP300 in regulating ferroptosis, we overexpressed EP300 in PDAC cells (Figure S2A). The overexpression of EP300 cDNA increased sensitivity to RSL3 compared to the control cDNA (Figures S2B-S2F). Indeed, the facilitation of EP300 promoted RSL3-induced ferroptotic events, including lipid ROS production (Figure S2G), MDA generation (Figure S2H), and iron accumulation (Figure S2I-S2J). These findings further suggest that EP300 is a positive regulator of ferroptosis.

### EP300 mediates HSPA5 acetylation in ferroptosis

**Figure 3:**
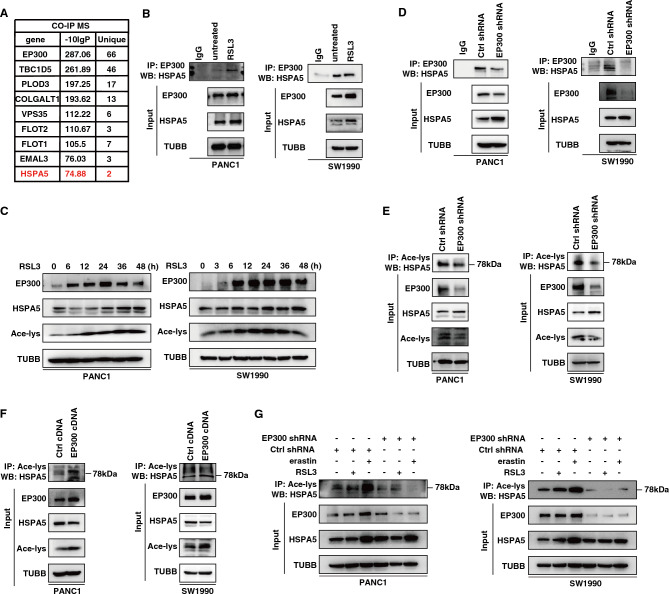
Interaction of HSPA5 and histone acetyltransferase EP300. (**A**) The form showed EP300-interacting proteins obtained by Co-IP MS. (**B**) Immunoprecipitation (IP) analysis of the interaction between HSPA5 and EP300 following treatment with RSL3 for 12 h in indicated PDAC cells. (**C**) Western blot analysis of HSPA5 and Acetyl lysine protein expression in PANC1 and SW1990 cells following treatment with RSL3 (1 μM) for 0–48 h. (**D**) Immunoprecipitation (IP) analysis of the interaction between HSPA5 and EP300 in the EP300 knockdown cell lines. (**E**) HSPA5 acetylation was analyzed by immunoprecipitation with an anti-acetyl-lys antibody followed by western blotting for EP300 knockdown cell lines. (**F**) HSPA5 acetylation was analyzed by immunoprecipitation with an anti-acetyl-lys antibody followed by western blotting for EP300 overexpression in cell lines. (**G**) HSPA5 acetylation was analyzed by immunoprecipitation after treated with erastin (10 μM, 12 h) or RSL3 (1 μM, 12 h) in the EP300 knockdown cell lines.

To understand how EP300 expression enables cell sensitivity for ferroptosis, we performed co-immunoprecipitation (Co-IP) mass spectrometry analysis of EP300-binding proteins in SW1990 cells in the absence or presence of RSL3 treatment. To mitigate the effects of potential contamination and/or non-specific binding of the IgG portion of the anti-EP300 antibody (αEP300), candidates for EP300-interacting proteins were screened using the following criteria: (1) Proteins identified in the αEP300-IP group need to be excluded from those identified in the IgG-IP negative control group; (2) Proteins with at least 2 unique peptides; (3) Protein confidence score (-10lgP) greater than 70. In addition to EP300 itself, a total of 8 proteins (TBC1 domain family member 5 [TBC1D5], procollagen-lysine,2-oxoglutarate 5-dioxygenase 3 [PLOD3], collagen beta (1-O) galactosyltransferase 1 [COLGALT1], VPS35 retromer complex component (VPS35), flotillin 1 [FLOT1], flotillin 2 [FLOT2], EMAP like 3 [EML3] and HSPA5) were identified as binding to EP300 in response to RSL3 (Fig. 3A, Supplementary Data 1).

Since HSPA5 has been previously identified as a regulator of ferroptosis^15, 24^, we next focused on the effect of EP300 on HSPA5. As expected, Co-IP confirmed the interaction between HSPA5 and EP300 was increased by RSL3 in PANC1 and SW1990 cells (Fig. 3B). Western blotting observed a correlation between the upregulation of HSPA5 and the total acetylation level of the protein (Fig. 3C). Importantly, the knockdown of EP300 in PANC1 and SW1990 cells reduced protein binding (Fig. 3D) and the basal acetylation level of HSPA5 (Fig. 3E). In contrast, the overexpression of EP300 increased HSPA5 acetylation at baseline (Fig. 3F). Furthermore, RSL3 and erastin increased the acetylation of HSPA5 (Figure S3A-S3B). The genetic or pharmacological inhibition of EP300 suppressed the increased acetylation of HSPA5 by RSL3 and erastin (Fig. 3G, S3C), further supporting that HSPA5 is an acetylated substrate protein for EP300 during ferroptosis.

EP300 is not only an acetyltransferase, but also a polyubiquitin ligase enzyme^25^. We found that HSAP5 protein was increased in EP300-knockdown cell lines (Figure S3D). In eukaryotic cells, protein homeostasis requires selective degradation by the ubiquitin–proteasome system (UPS) and autophagic pathway^26^. The proteasome inhibitor MG132, but not the autophagy inhibitor bafilomycin A1, caused the accumulation of HSPA5 protein (Figure S3E), indicating that the UPS pathway mediates HSPA5 degradation. Since HSPA5 inhibits ferroptosis by preventing GPX4 degradation^15^, we also examined the effect of EP300 inhibition on GPX4 expression during ferroptosis. Western blotting showed that genetic or pharmacological inhibition of EP300 reversed the decrease in GPX4 caused by RSL3 and erastin **(**Figure S3F-S2G**)**. In addition, the overexpression of EP300 promoted GPX4 degradation through HSPA5 acetylation (Figure S3H). These findings suggest that the EP300-HSPA5 complex can regulate GPX4 protein levels during ferroptosis.

### Acetylation of HSPA5 at K353 mediates ferroptosis

**Figure 4:**
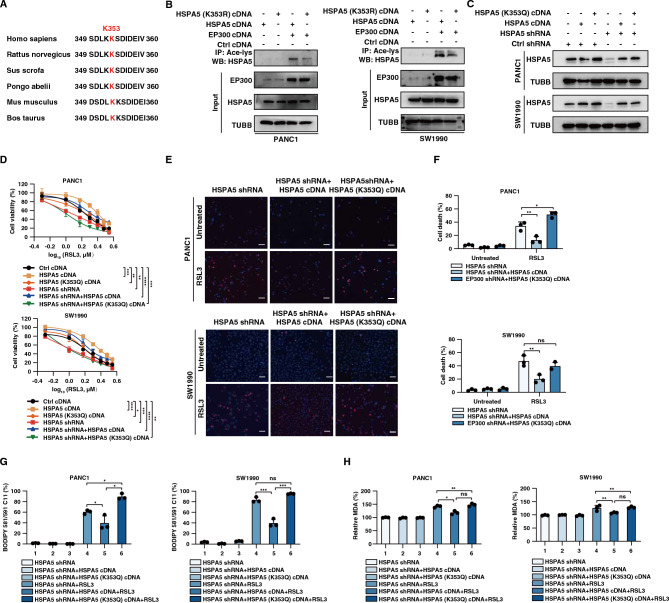
EP300 acetylates HSPA5 at K353. (**A**) Alignment of HSPA5 amino acid sequence from various species. red shading indicates the conserved K353. (**B**) SW1990 and PANC1 cells were transfected with HSPA5 wild type or HSPA5/K353R mutant construct in the presence or absence of EP300 expressing vector, cell lysates were harvested for IP and western blot assays. (**C**) Western blot analysis of HSPA5 protein expression in HSPA5-knockdown PANC1 and SW1990 cells which transfected with HSPA5 or HSPA5-K353Q. (**D**-**F**) Measuring cell survival by cell count kit 8, cell death by PI/hoechst33342 staining in the indicated cells following treatment with RSL3 (1 μM) for 24 h in the presence or absence of HSPA5/K353Q mutant expressing cell. Scale bar: 100 μm. (**G**-**H**) Measuring lipid peroxidation and oxidative stress using an MDA assay kit or BODIPY C11 probe in indicated cells following treatment with RSL3 (1 μM) for 24 h in the presence or absence of HSPA5/K353Q mutant expressing cell.

A previous study found that EP300 can acetylate HSPA5 through the K353 site^27^. We found that the K353 site was highly conserved across species (Fig. 4A). We next examine whether K353 is a key site for HSPA5 acetylation during ferroptosis. We generated the K353R mutant. Compared with WT-HSPA5, HSPA5-K353R showed markedly reduced acetylation (Fig. 4B). Next, we examined the ability of WT and K353Q mutants to restore the phenotype of HSPA5 knockdown cells in ferroptosis (Fig. 4C). After assaying for cell death, lipid ROS and MDA, we demonstrated that WT-HSPA5, but not HSPA5-K353R, reversed the ferroptotic phenotype in HSPA5-knockdown PDAC cells (Fig. 4D–H). These data support that K353 is the main site of HSPA5 acetylation during ferroptosis.

### HSPA5 is deacetylated by HDAC6

**Figure 5:**
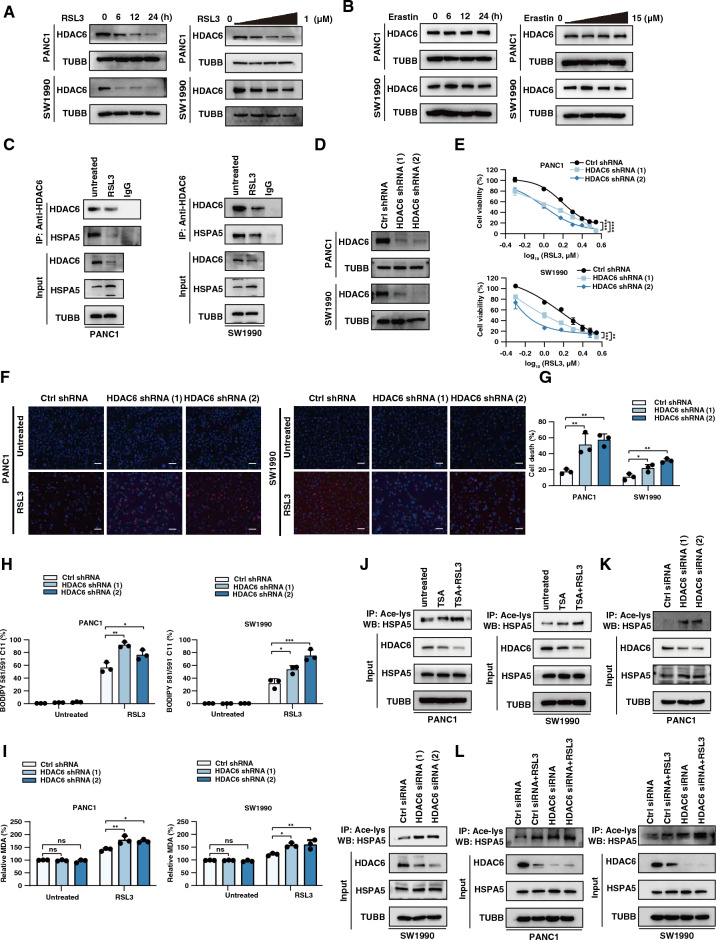
HSPA5 is deacetylated by HDAC6. (**A**) Western blot analysis of HDAC6 protein expression in PANC1 and SW1990 cells following treatment with RSL3 (0–1 μM) for 0–24 h. (**B**) Western blot analysis of HDAC6 protein expression in PANC1 and SW1990 cells following treatment with erastin (0–15 μM) for 0–24 h. (**C**) Immunoprecipitation (IP) analysis of the interaction between HSPA5 and HDAC6 following treatment with RSL3 (1 μM) for 12 h. (**D**) Western blot analysis of HDAC6 protein expression in HDAC6-knockdown PANC1 and SW1990 cells. (**E**–**G**) Measuring cell survival by cell count kit 8, cell death by PI/hoechst33342 staining in the indicated cells following treatment with RSL3 (1 μM) for 24 h. Scale bar: 100 μm. (**H**-**I**) Measuring lipid peroxidation and oxidative stress using an MDA assay kit or BODIPY C11 probe in indicated cells following treatment with RSL3 (1 μM) for 24 h. (**J**) Immunoprecipitation analysis of HSPA5 acetylation using anti-acety-lys treatment with TSA (0.1 mM) or RSL3 (1 μM) for 12 h. (**K**) Immunoprecipitation analysis of HSPA5 acetylation in HDAC6 knockdown PDAC cells. (**L**) Immunoprecipitation analysis of HSPA5 acetylation following treatment with RSL3 (1 μM) in HDAC6 knockdown PDAC cells. PDAC, pancreatic ductal adenocarcinoma; PI, propidium iodide; Ace-lys, acetylated lysine.

HDACs are a class of enzymes that can remove acetyl groups in various proteins. HDAC6 has previously been reported as a deacetylase of HSPA5 in breast cancer cells^28^. RSL3 but not erastin dose and time-dependently decreased HDAC6 protein expression in human PDAC cells (Fig. 5A–B). Next, we investigated whether HDAC6 plays the opposite role of EP300 in regulating HSPA5 acetylation as well as ferroptosis sensitivity. Immunoprecipitation analysis confirmed the interaction between HDAC6 and HSPA5 in PDAC cells in the absence or presence of RSL3 treatment (Fig. 5C). Functionally, the knockdown of HDAC6 increased RSL3-induced ferroptosis (Fig. 5D–I). TSA, an inhibitor of the HDAC family, in combination with RSL3 significantly increased HSPA5 acetylation (Fig. 5J). The knockdown of HDAC6 increased acetylation of HSPA5 by RSL3 (Fig. 5K–L). In conclusion, HDAC6 inhibits ferroptosis in human PDAC cells.

## Discussion

Ferroptosis, a regulated necrosis caused by disruption of antioxidant defenses and ROS production has recently aroused extensive attention in translational medicine^29^. The mechanism by which ferroptosis inhibits tumor cell growth is different from apoptosis and necroptosis. Studies have shown that ferroptosis inducers cooperate with chemotherapeutic drugs to exert anti-tumor activity, and inhibition of ferroptosis has also become a key reason for anti-tumor drug resistance^30^. The present results suggest that the upregulation of EP300 is a positive regulator of ferroptosis in PDAC cells. EP300 and HDAC6 interacted with HSPA5 in ferroptosis. Increasing EP300 and decreasing HDAC6 protein expression promoted lipid peroxidation in ferroptosis by directly enhancing HSPA5 acetylation. These findings also led to a significant increase in the anticancer activity of RSL3 in combination with HDAC6 inhibitors in the treatment of PDAC by inducing ferroptosis. In contrast, the EP300 inhibitor C646 reversed RLS3-induced ferroptosis.

Acetylation occurs by the transfer of an acetyl group from acetyl-CoA to the ε-amino side chain of lysine by acetyltransferases, a process that can be reversed by deacetylases^30^. Despite a clear link between histone acetylation and cancer development^32^, the contribution of aberrant acetylation to ferroptotic cancer cell death has not been well studied. We found that the acetyltransferase EP300 and the deacetylase HDAC6 play critical roles in controlling the acetylation of HSPA5 in PDAC cells, resulting in changes in ferroptosis sensitivity. TSA is a widely used HDAC inhibit or with broad epigenetic activity that alters the ability of DNA transcription factors to access DNA molecules within chromatin^33^. Early studies indicate that TSA prevents the formation of the HSPA5-caspase-7 complex, leading to an increased etoposide-induced apoptosis in breast cancer cells^34^. Our current study demonstrates that RSL3 in combination with TSA significantly enhanced HSAP5 acetylation levels, leading to an increased RSL3-induced ferroptosis in PDAC cells. Thus, TSA has a great potential for PDAC treatment.

The unfolded protein response (UPR) is initiated by three ER-resident transmembrane proteins that function as sensors of protein-folding stress: inositol-requiring protein 1α (ERN1), PRKR-like ER kinase (EIF2AK3) and activating transcription factor 6 (ATF6). Under normal conditions, HSPA5 binds to these ER sensors to inhibit their activation^35^. Under ferroptotic conditions, HSPA5 can form a complex with GPX4 and inhibit GPX4 protein degradation^15^. Here, we demonstrated that acetylated HSPA5 has a stronger role in promoting RSL3-induced ferroptosis by hijacking GPX4 to accelerate lipid peroxidation. Persistent ER stress and protein misfolding-triggered ROS cascades involve many regulators. For example, endoplasmic reticulum oxidoreductin-1 (ERO1) is closely related to protein load in the ER, triggering ROS generation during ER stress^36^. It will be interesting to understand how acetylation of HSPA5 affects the folding function of the endoplasmic reticulum, leading to oxidative stress. Monitoring changes in ER redox status also requires observing calcium fluxes, which may affect membrane repair during ferroptosis. Nevertheless, the function of specialized ER stress sensors in ferroptosis may depend on the status of HSPA5 and its partners.

In summary, we demonstrated that a new PTM mechanism deciding ferroptosis sensitivity. Specifically, EP300-mediated acetylation of HSPA5 enhanced HSPA5-mediated inhibition of GPX4, resulting in excessive lipid peroxidation and ferroptosis. These findings may provide clues for new strategies to target PTM machinery in pancreatic cancer. (Supplementary Information 1 and 4).

### Supplementary Information

Supplementary Information 1.

Supplementary Figures.

Supplementary Tables.

Supplementary Figures.

## Data Availability

The datasets generated and/or analysed during the current study are available in the “PRIDE” repository. Website: https://www.ebi.ac.uk/pride/archive/projects/PXD039998/private Username: reviewer_pxd039998@ebi.ac.uk, Password: kJGVEr2Z.
