# Supplementary material for: EP300 promotes ferroptosis via HSPA5 acetylation in pancreatic cancer
Source: Sci Rep. 2023 Sep 11;13:15004. doi: 10.1038/s41598-023-42136-8 (PMC10495396; doi:10.1038/s41598-023-42136-8)
Supplement: Supplementary file 2 — Supplementary Figures. [file 41598_2023_42136_MOESM2_ESM.pdf]

Figure S1

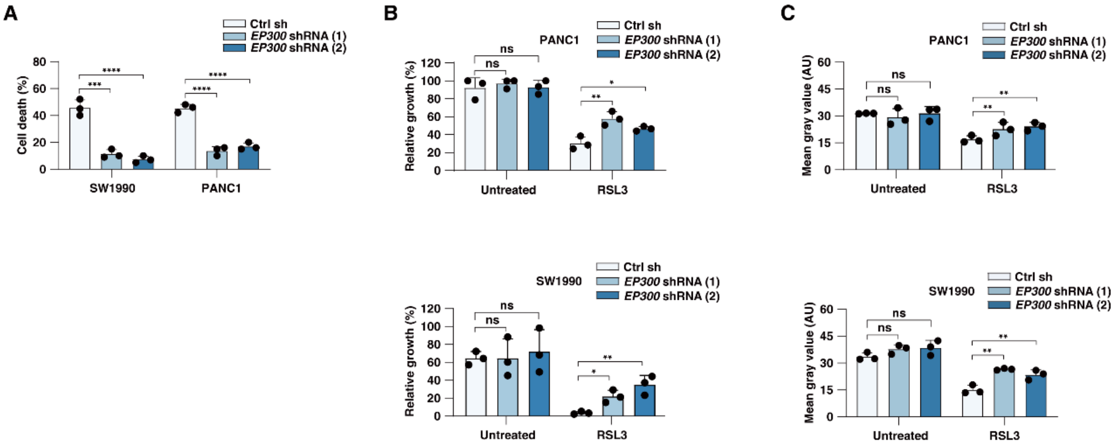

Figure S1. EP300 positively regulates ferroptosis. (A) Cell death by PI/hoechst33342 staining in the indicated cells following treatment with RSL3 (1 μM) for 24 h. Scale bar: 100 μm. (B) Colony formation assay in indicated PDAC cells following treatment with RSL3 (1 μM) for 24 h; then an equal amount of surviving cells was re-cultured in 6-well plates for 14 days. (C) Cellular iron accumulation level was assayed by PGSK (Phen Green SK) probe, Fluorescence intensity was observed under the fluorescence microscope. Scale bar: 100 μm.

Figure S2

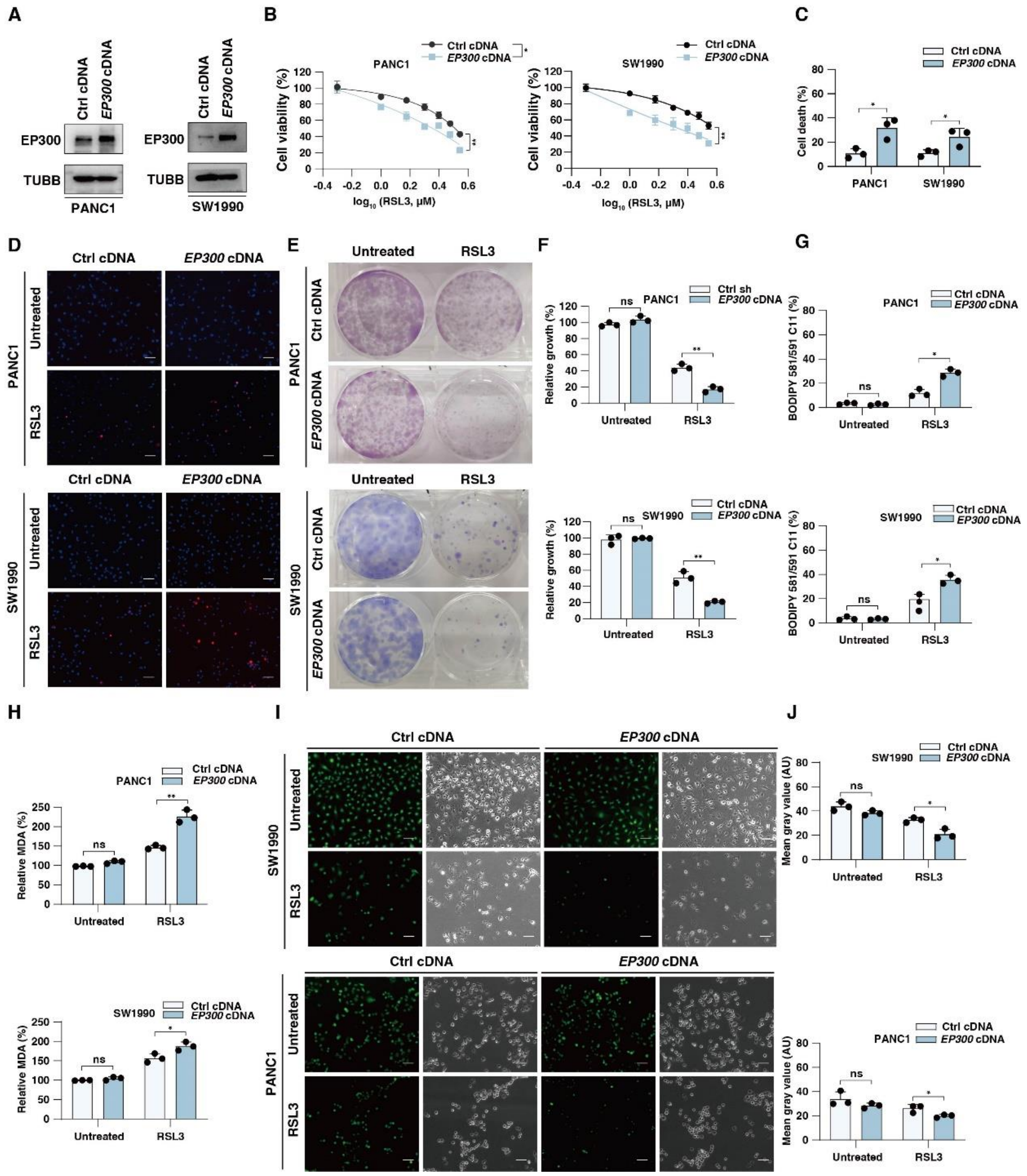

Figure S2 EP300 positively regulates ferroptosis. (A) Western blot analysis of EP300 protein expression in EP300-overexpression in PANC1 and SW1990 cells. (B) Measuring cell survival by cell count kit 8 following treatment with RSL3 (1 μM) for 24 h. (C-D) Cell death by PI/hoechst33342 staining in the indicated cells following treatment with RSL3 (1 μM) for 24 h. Scale bar: 100 μm. (E-F) Colony formation assay in indicated PDAC cells following treatment with RSL3 (1 μM) for 24 h; then an equal amount of surviving cells was re-cultured in 6-well plates for 14 days. (G) The lipid ROS level was assessed by flow cytometry using a C11-BODIPY probe in indicated cells following treatment with RSL3 (1 μM) for 24 h. (H) Measuring oxidative stress using an MDA assay kit in indicated cells following treatment with RSL3 (1 μM) for 24 h. (I-J) Cellular iron accumulation level was assayed by PGSK (Phen Green SK) probe, Fluorescence intensity was observed under the fluorescence microscope. Scale

bar: 100 μm.

Figure S3

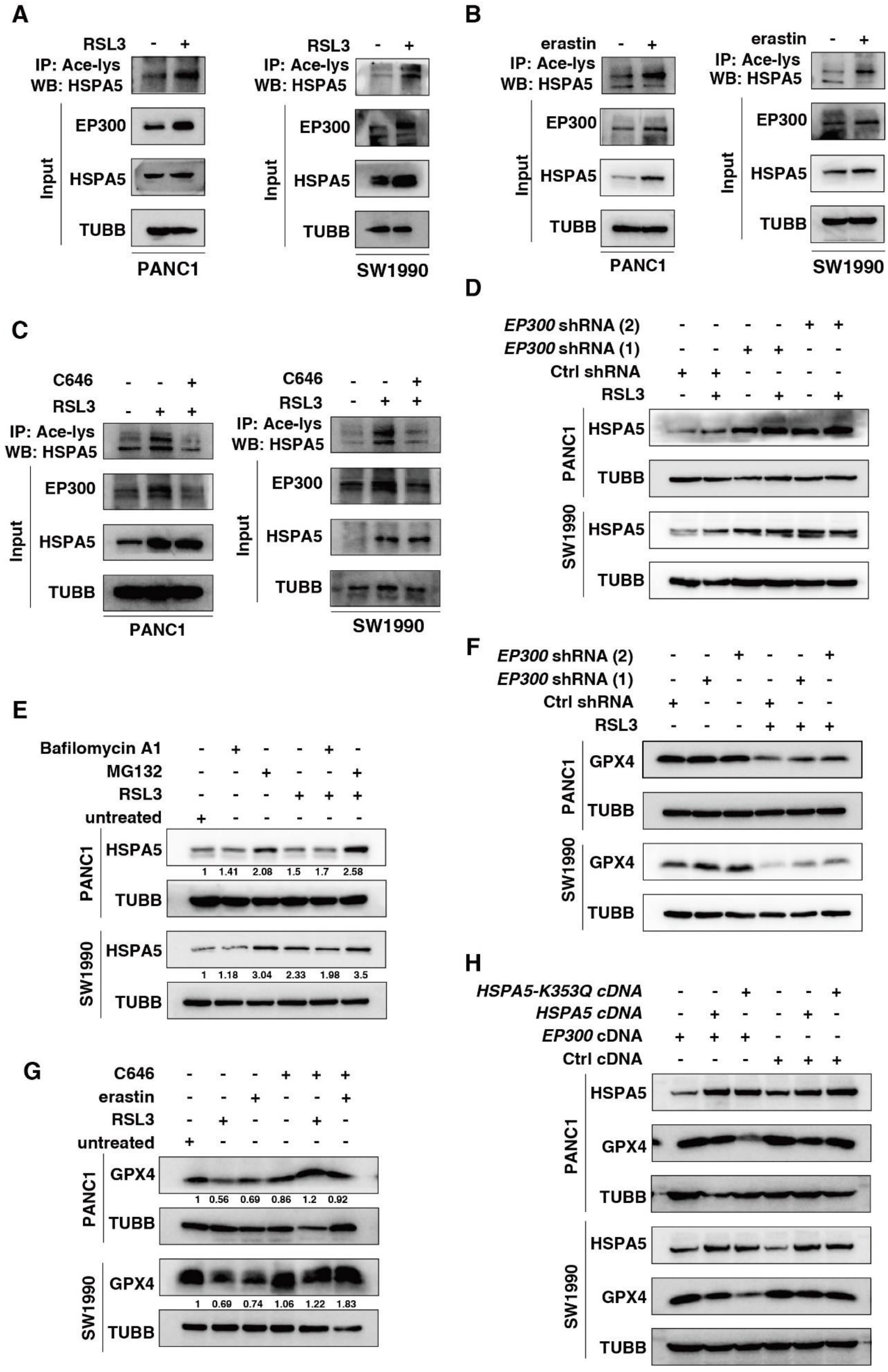

Figure S3. Interaction of HSPA5 and histone acetyltransferase EP300. (A, B) HSPA5 acetylation was analyzed by immunoprecipitation with an anti-acetyl-lys antibody followed by western blotting in PANC1 and SW1990 cells treated with RSL3 (1 μM) or erastin (10 μM) for 12 h. (C) HSPA5 acetylation was analyzed by immunoprecipitation with an anti-acetyl-lys antibody followed by western blotting in PANC1 and SW1990 cells treated RSL3 (1 μM) and C646 (40 μM) for 12 h. (D) Western blot analysis of HSPA5 protein expression in the EP300 knockdown cell lines treated RSL3 (1 μM) for 24 h. (E) Western blot analysis of HSPA5 protein expression in PANC1 and SW1990 cells treated with Bafilomycin (0.5 μM) or MG132 (0.25 μM) and RSL3 (1 μM) for 24 h. (F) Western blot analysis of GPX4 protein expression in the EP300 knockdown PANC1 and SW1990 cells following treatment with RSL3 (1 μM) for 24 h. (G) Western blot analysis of GPX4 protein expression in PANC1 and SW1990 cells following treatment with RSL3 (1 μM) or erastin (10 μM) and C646 (40 μM) for 24 h. (H) SW1990 and PANC1 cells were transfected with HSPA5 wild type or HSPA5/K353Q mutant construct in the vector or EP300 expressing vector, cell lysates were harvested for western blot.
