# Supplementary material for: EP300 promotes ferroptosis via HSPA5 acetylation in pancreatic cancer
Source: Sci Rep. 2023 Sep 11;13:15004. doi: 10.1038/s41598-023-42136-8 (PMC10495396; doi:10.1038/s41598-023-42136-8)
Supplement: Supplementary file 3 — Supplementary Tables. [file 41598_2023_42136_MOESM3_ESM.pdf]

Table S1: Gene cloning sequences

RNAi and gene transfection		
**Gene name**	**Source**	**Sequence**
EP300 cDNA	Genechem	F: 5’-CCCAACTTTGTGCCA ACCGGTCGCCACCATGGC CGAGAATGTGGTGGAAC CGGGG- 3’ R: 5’-CACACATTCCACAGG AATTCTAGGATCCACGCGG AACCAGATCCGATTTTG- 3’
EP300-shRNA (1)		F: 5’-CCGGCAATTCCGAGA CATCTTGAGACTCGAGTCT CAAGATGTCTCGGAATTGT TTTTG- 3’ R: 5’-AATTCAAAAACAATT CCGAGACATCTTGAGACTC GAGTCTCAAGATGTCTCGG AATTG- 3’
EP300-shRNA (2)		F: 5’-CCGGCCCGGTGAACT CTCCTATAATCTCGAGATT ATAGGAGAGTTCACCGGG TTTTTG- 3’ R: 5’-AATTCAAAAACCCG GTGAACTCTCCTATAATCT CGAGATTATAGGAGAGTTC ACCGGG- 3’
HSPA5 cDNA	Genechem	F: 5’-CCGGAATTCGCCACC ATGAAGCTCTCCCTGGTG G- 3’ R: 5’-AAGGAAAAAAGCGG

		CCGCCAACTCATCTTTTTC TGCTG- 3’
HSPA5 K353R		F: 5’-GATTTGAAGAgGTCT GATATTGATGAAATTGTTC TTGTT -3'<br>R: 5'-TCAGACcTCTTCAAA TCAGAATCTTCCAACACT -3'
HSPA5 K353Q		F: 5'-GATTTGAAGcAGTCT GATATTGATGAAATTGTT CTTGTT -3'<br>R: 5'-TCAGACTgCTTCAAAT CAGAATCTTCCAACACTT-3'
HDAC6 siRNA (1)	Genepharma	F: 5'-CCAAGGAUAUACCA UCAAUTT-3'<br>R: 5'-AUUGAUGGUAUAUC CUUGGTT-3'
HDAC6 siRNA (2)	Genepharma	F: 5'-CCGUGAGAGUUCCA ACUUUTT-3'<br>R: 5'-AAAGUUGGAACUCU CACGGTT-3'
HDAC6 shRNA (1)		F: 5'-CCGGCCTCACTGATC AGGCCATATTCTCGAGAA TATGGCCTGATCAGTGAG GTTTTTG-3'<br>R: 5'- AATTCAAAAACCTCA CTGATCAGGCCATATTCTC GAGAATATGGCCTGATCAG

		TGAGG-3'
HDAC6 shRNA (2)		5'- CCGGCATCCCATCCTG AATATCCTTCTCGAGAAG GATATTCAGGATGGGATG TTTTTG-3'
		5'- AATTCAAAAACATCCC ATCCTGAATATCCTTCTCG AGAAGGATATTCAGGATG GGATG-3'
HSPA5 shRNA (1)	Addgene	5'- GTACCGGAGATTCAG CAACTGGTTAAAGCTCGA GCTTTAACCAGTTGCTGA ATCTTTTTTTG-3'
HSPA5 shRNA (2)	Addgene	5'- CCGGGAAATCGAAA GGATGGTTAATCTCGAGA TTAACCATCCTTTCGATT TCTTTTTG-3'
