# Supplementary material for: EP300 promotes ferroptosis via HSPA5 acetylation in pancreatic cancer
Source: Sci Rep. 2023 Sep 11;13:15004. doi: 10.1038/s41598-023-42136-8 (PMC10495396; doi:10.1038/s41598-023-42136-8)

Figure 1A

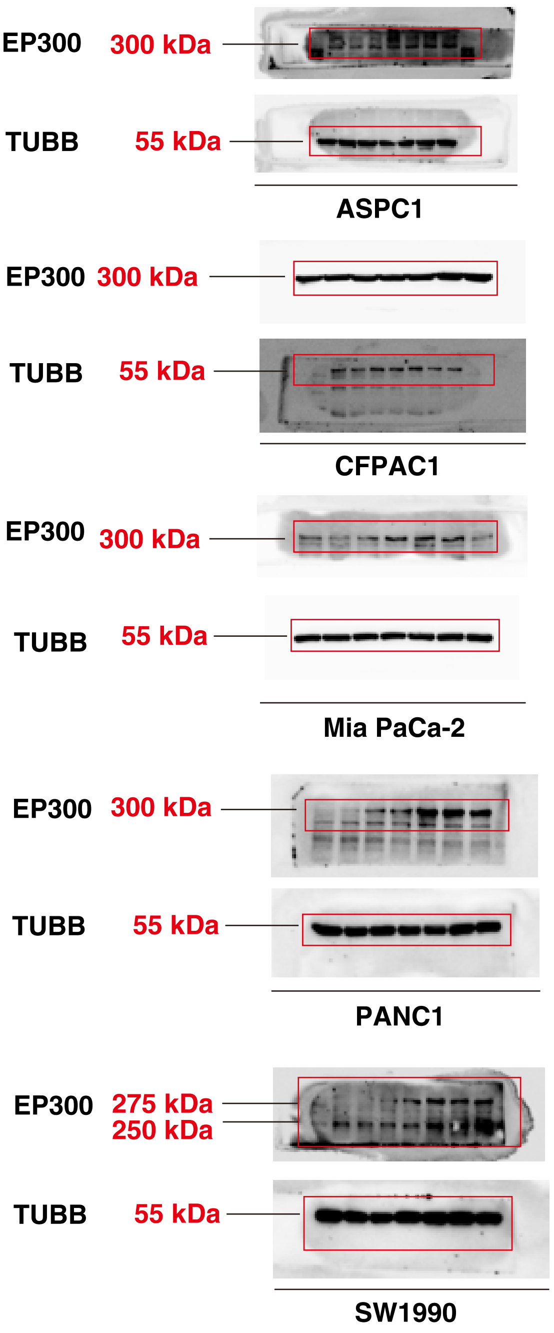

Figure 1C-1D

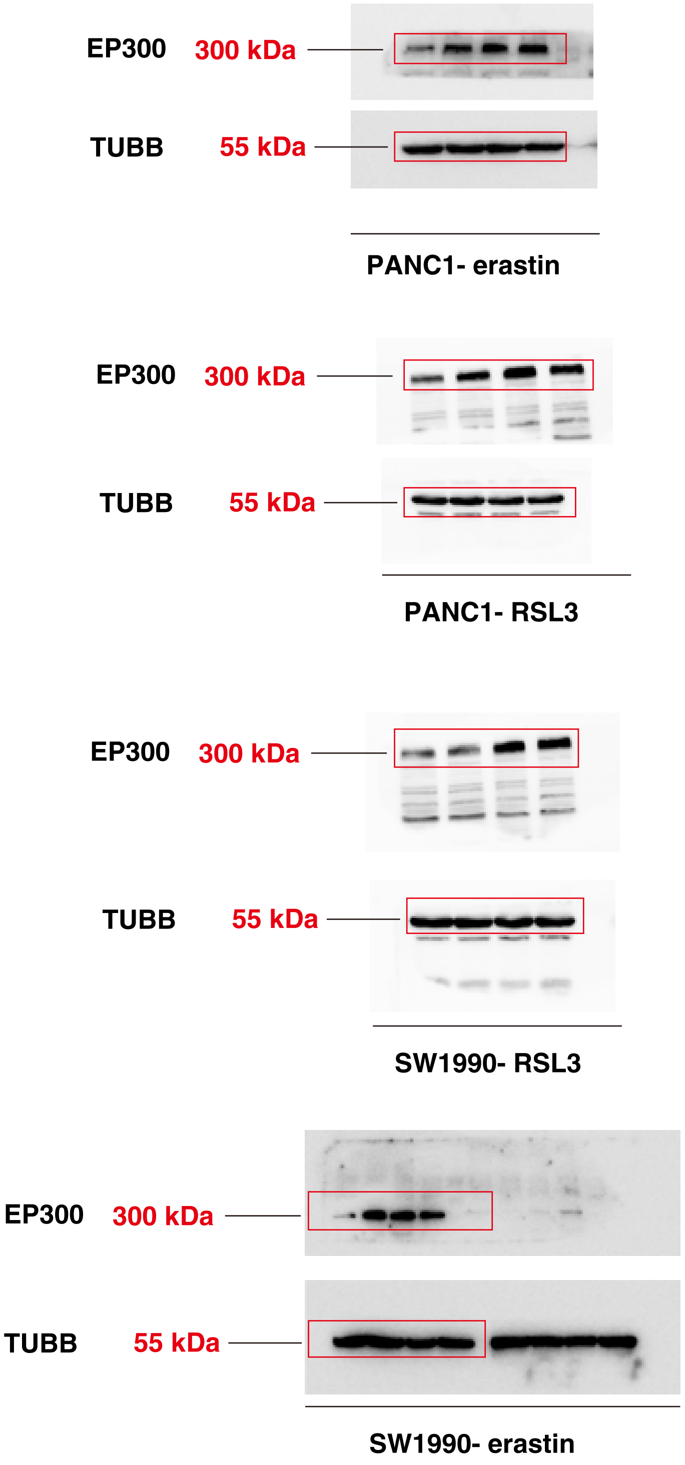

Figure 2A

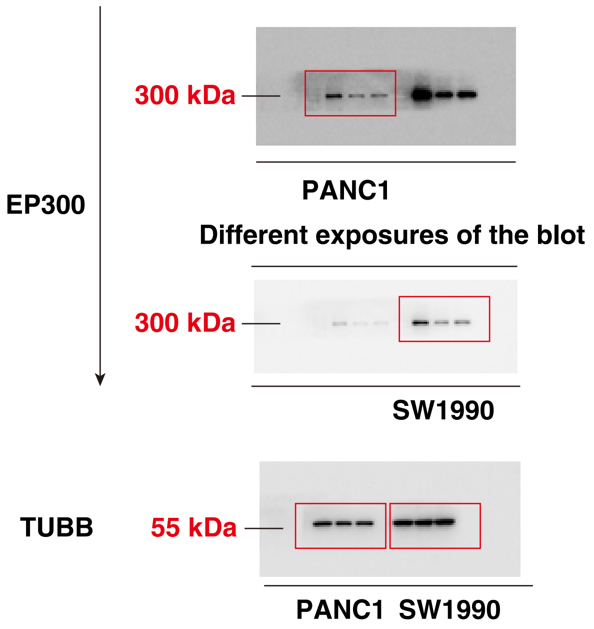

Figure 3C

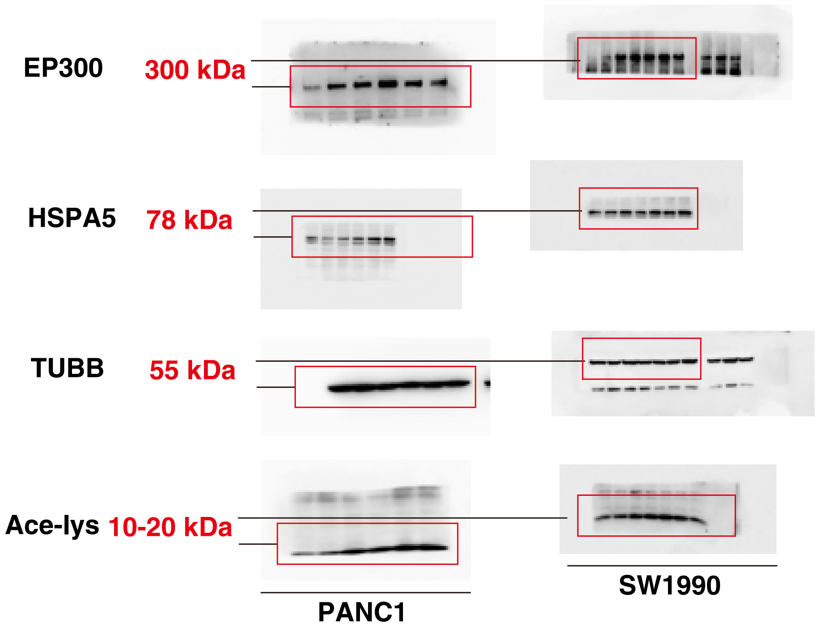

Figure 3B

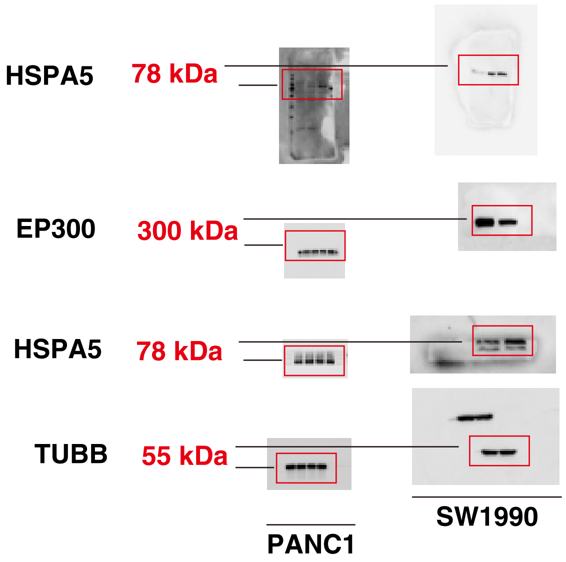

Figure 3D

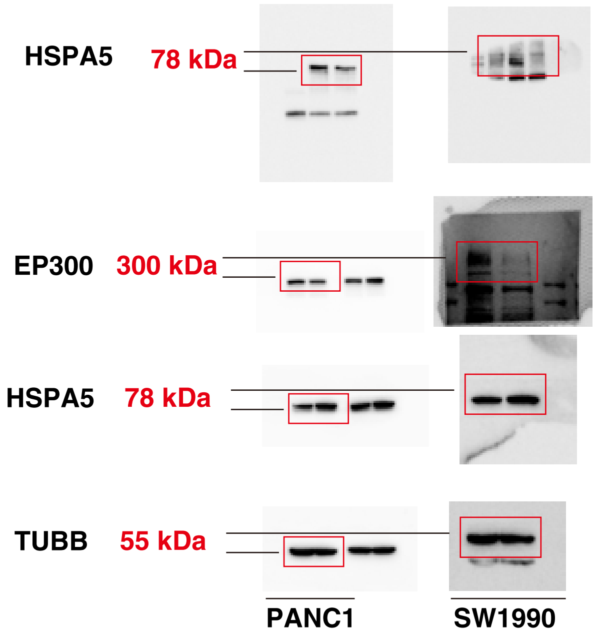

Figure 3E

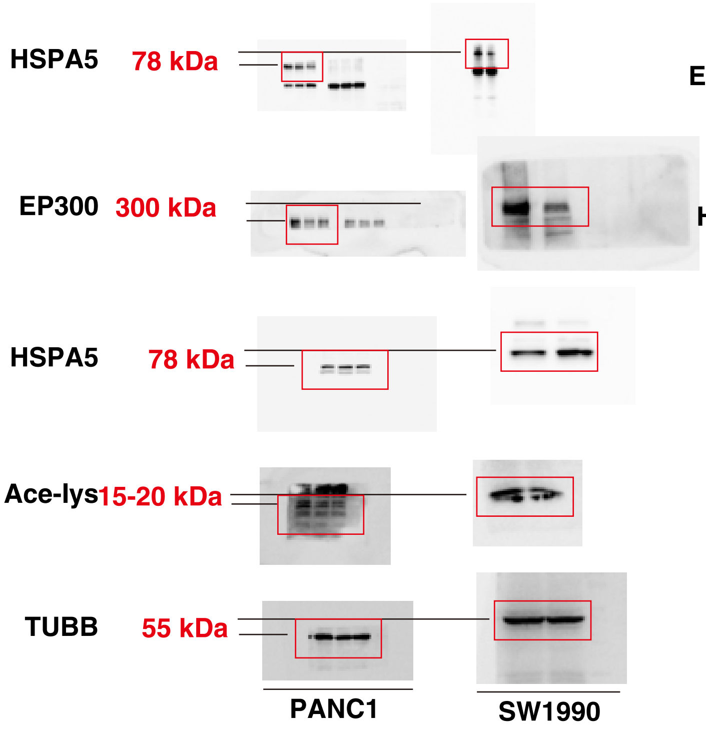

Figure 3F

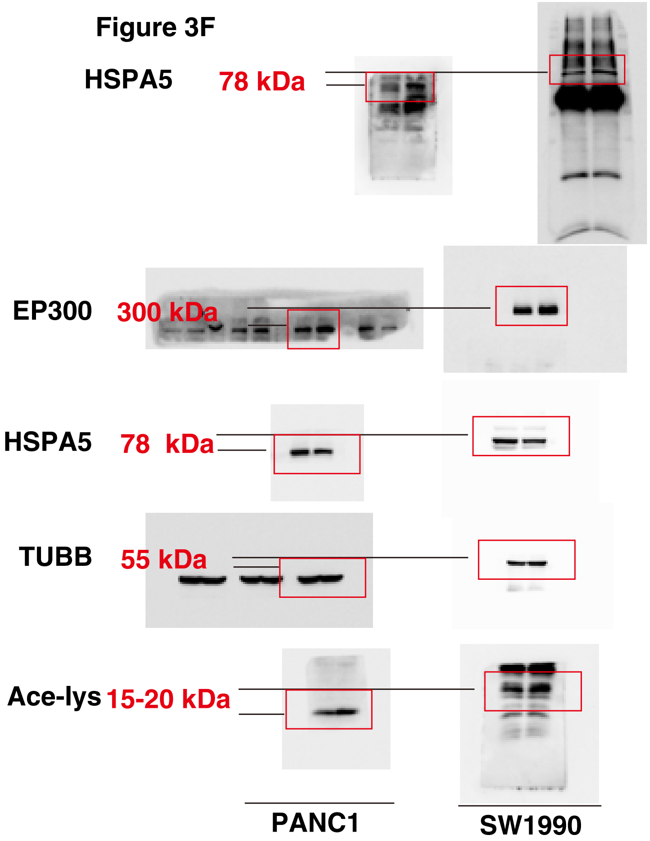

Figure 3G

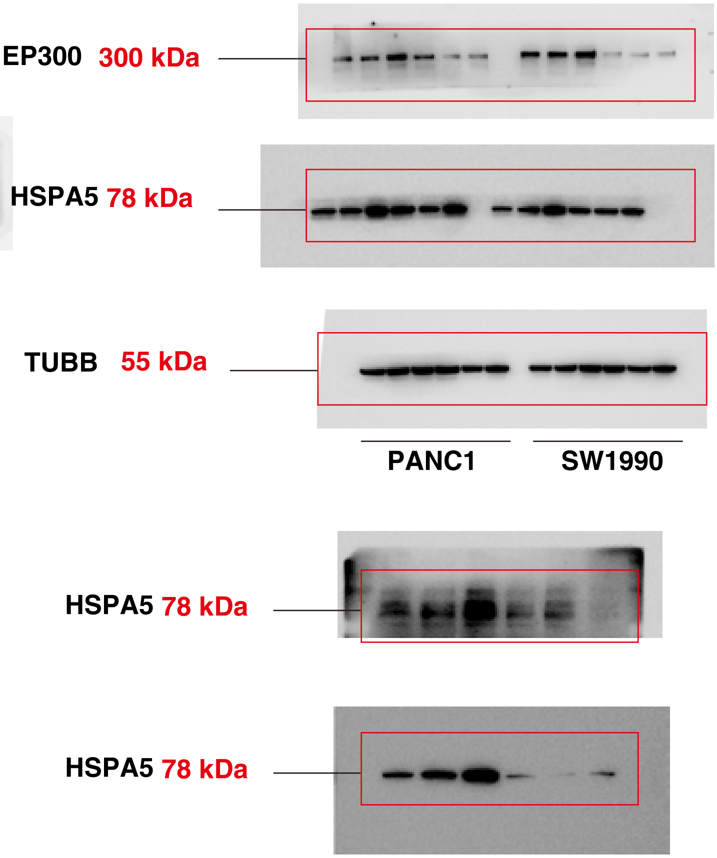

Figure S2A

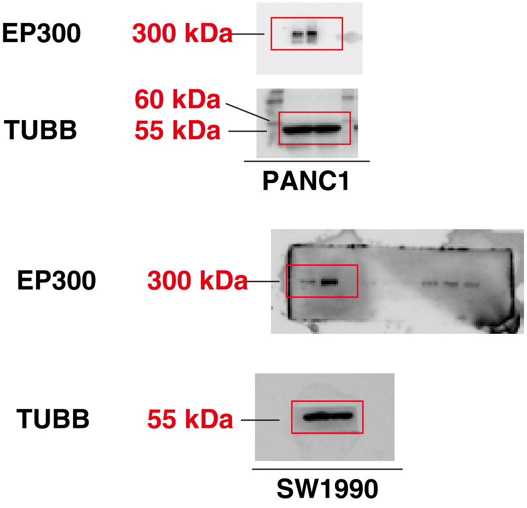

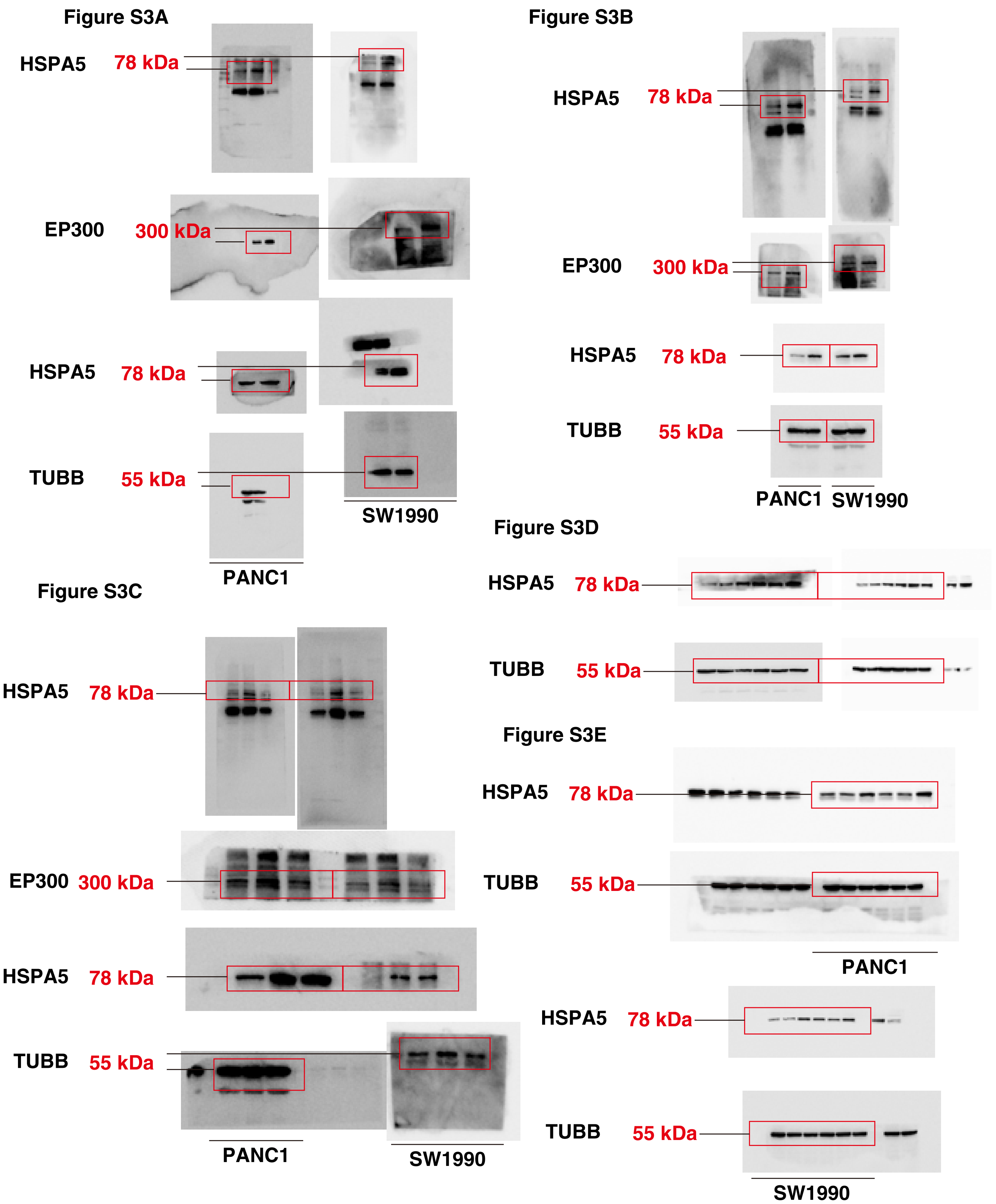

Figure S3A
HSPA5 78 kDa
EP300 300 kDa
HSPA5 78 kDa
TUBB 55 kDa
SW1990
PANC1
Figure S3B
HSPA5 78 kDa
EP300 300 kDa
HSPA5 78 kDa
TUBB 55 kDa
PANC1 SW1990
Figure S3C
HSPA5 78 kDa
EP300 300 kDa
HSPA5 78 kDa
TUBB 55 kDa
PANC1
SW1990
Figure S3D
HSPA5 78 kDa
TUBB 55 kDa
Figure S3E
HSPA5 78 kDa
TUBB 55 kDa
PANC1
HSPA5 78 kDa
TUBB 55 kDa
SW1990

Figure S3F

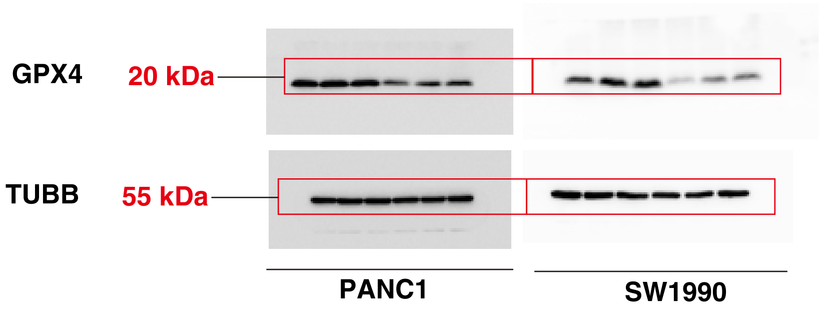

Figure S3G

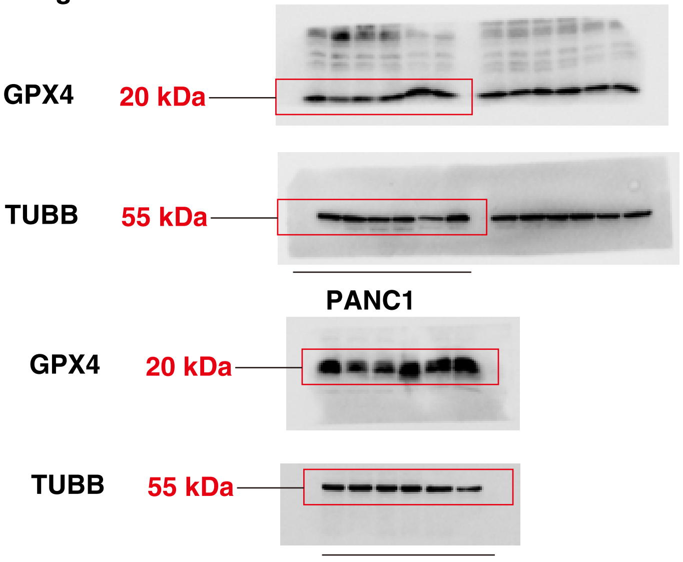

Figure S3H

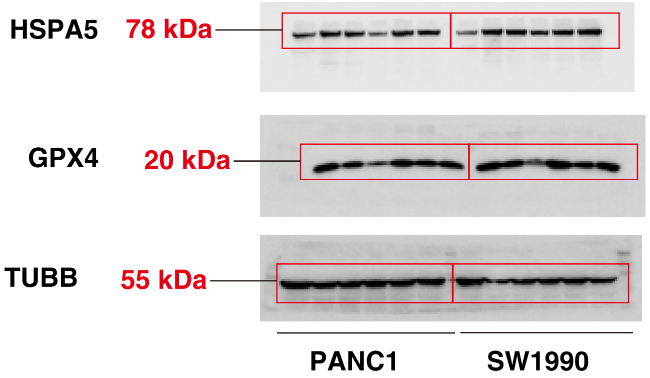

Figure 4B

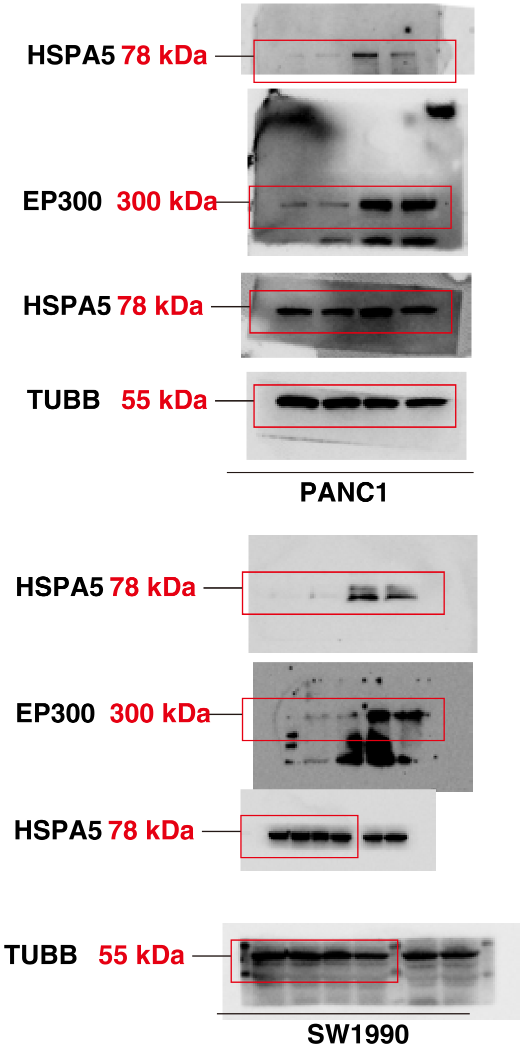

Figure 4C

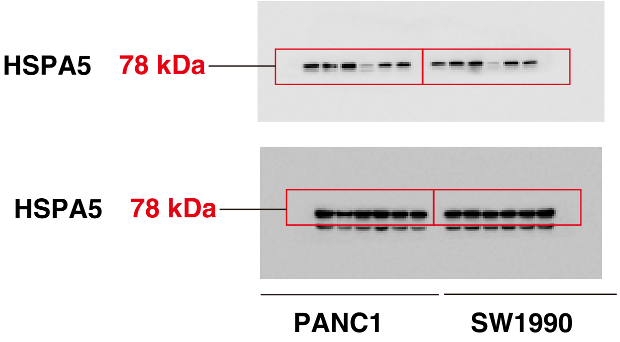

Figure 5A

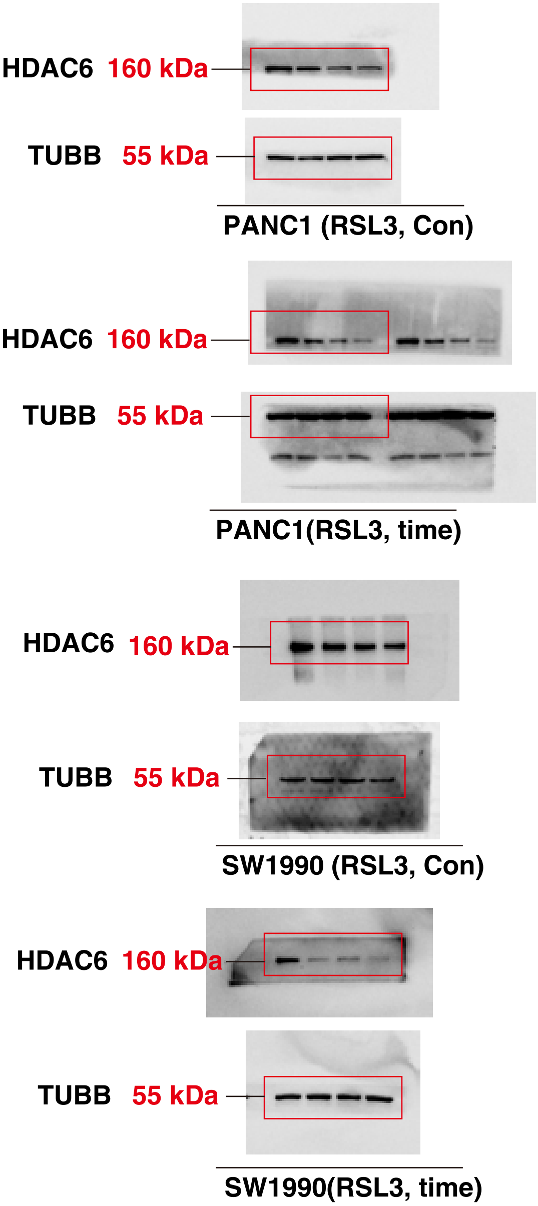

Figure 5B

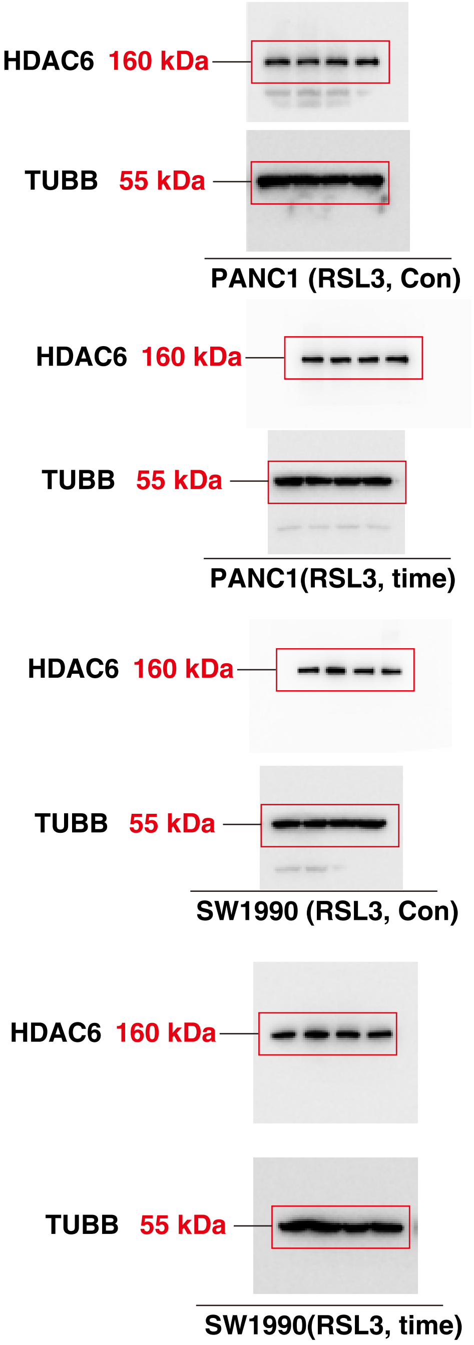

Figure 5C

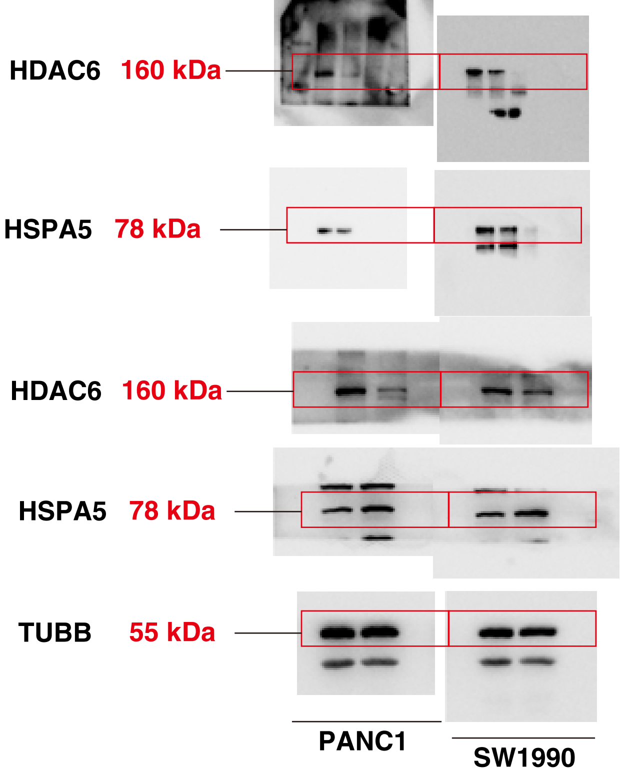

Figure 5D

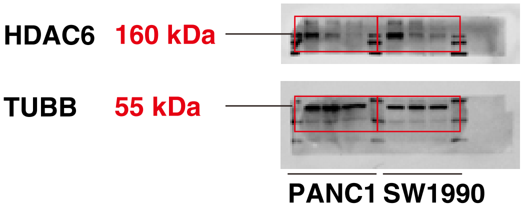

Figure 5J

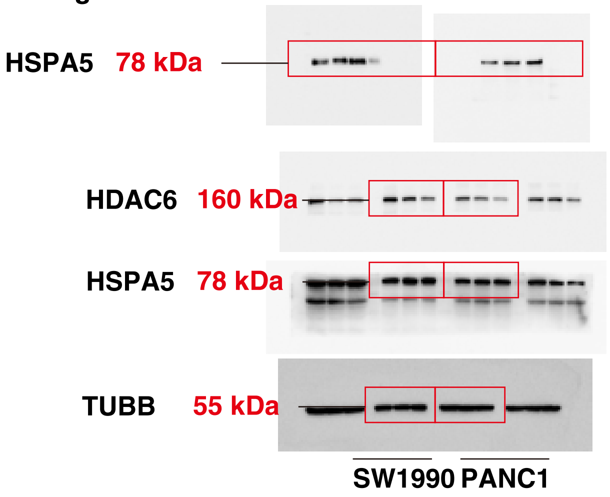

Figure 5K

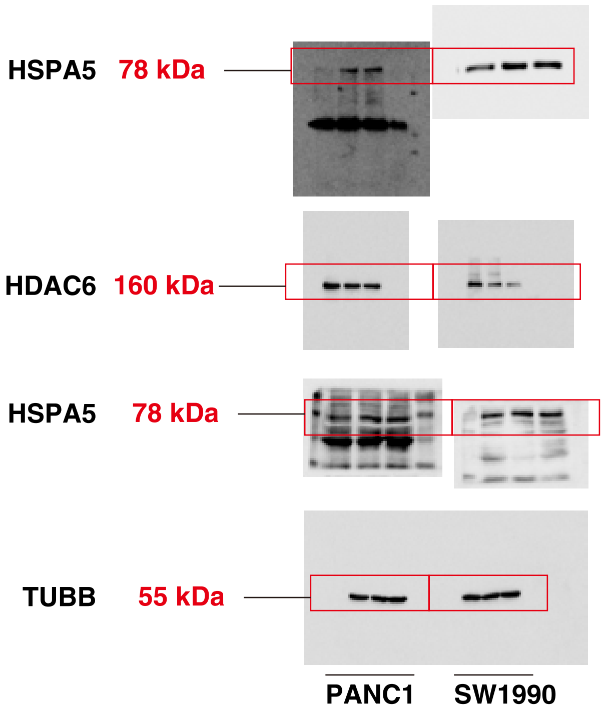

Figure 5L

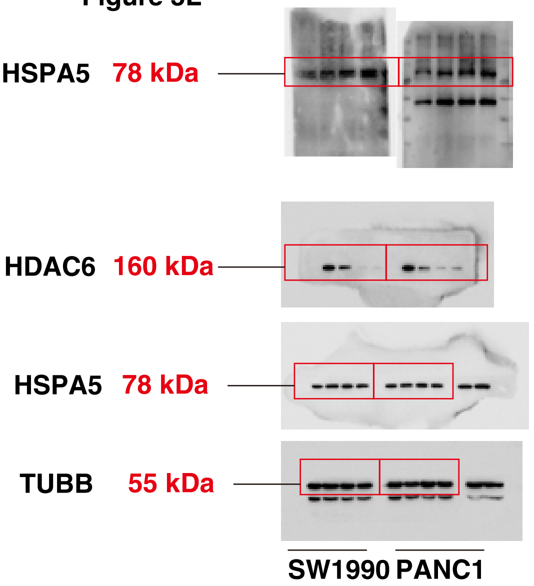

Supplement: Supplementary file 4 — Supplementary Figures. [file 41598_2023_42136_MOESM4_ESM.pdf]
